# Strong position-dependent effects of sequence mismatches on signal ratios measured using long oligonucleotide microarrays

**DOI:** 10.1186/1471-2164-9-317

**Published:** 2008-07-03

**Authors:** Catriona Rennie, Harry A Noyes, Stephen J Kemp, Helen Hulme, Andy Brass, David C Hoyle

**Affiliations:** 1 Biosciences Building, School of Biological Sciences, University of Liverpool, Crown Street, Liverpool, L69 7ZB, UK; 2 Faculty of Life Sciences, University of Manchester, Smith Building, Oxford Road, Manchester, M13 9PT, UK; 3 School of Computer Science, Kilburn Building, University of Manchester, Oxford Road, Manchester, M13 9PL, UK; 4 North West Institute of Bio-Health Informatics, School of Medicine, Stopford Building, Oxford Road, Manchester, M13 9PT, UK

## Abstract

**Background:**

Microarrays are an important and widely used tool. Applications include capturing genomic DNA for high-throughput sequencing in addition to the traditional monitoring of gene expression and identifying DNA copy number variations. Sequence mismatches between probe and target strands are known to affect the stability of the probe-target duplex, and hence the strength of the observed signals from microarrays.

**Results:**

We describe a large-scale investigation of microarray hybridisations to murine probes with known sequence mismatches, demonstrating that the effect of mismatches is strongly position-dependent and for small numbers of sequence mismatches is correlated with the maximum length of perfectly matched probe-target duplex. Length of perfect match explained 43% of the variance in log_2 _signal ratios between probes with one and two mismatches. The correlation with maximum length of perfect match does not conform to expectations based on considering the effect of mismatches purely in terms of reducing the binding energy. However, it can be explained qualitatively by considering the entropic contribution to duplex stability from configurations of differing perfect match length.

**Conclusion:**

The results of this study have implications in terms of array design and analysis. They highlight the significant effect that short sequence mismatches can have upon microarray hybridisation intensities even for long oligonucleotide probes.

All microarray data presented in this study are available from the GEO database [1], under accession number [GEO: GSE9669]

## Background

Microarrays are widely used for monitoring gene expression levels, using cDNA as a target, and for monitoring DNA copy number variations using genomic DNA as a target (Comparative Genomic Hybridisation CGH) [2-6]. Clearly sequence mismatches will affect the efficiency of hybridisation to probes spotted on microarrays and therefore the accuracy with which microarray based assays report the gene expression levels or genomic copy numbers. Understanding and quantifying the errors introduced by sequence mismatches is important, not only for handling microarray data, but also data from any high-throughput assay that makes use of duplex formation, for example sequence capture for regional sequencing [7-10]. The effects of sequence mismatches on hybridisations using short oligonucleotides (32 mers or shorter, such as the 25 mers used in Affymetrix GeneChips) have been reported previously [2,11-13], but we are not aware of any comprehensive experimental studies on the effects of sequence mismatches on duplex formation with longer oligonucleotides (50 mers to 100 mers). This was the objective of the current study. We integrated mouse CGH microarray data with the 8 million SNP that have been published for 15 inbred strains [14] in order to identify the effect of mismatches at each position in the probe on log_2 _signal ratio. This made it possible to characterise sequence mismatches that affect microarray hybridisation on a genome-wide scale.

### Overview of a microarray hybridisation

Nucleic acid probes are tethered to a solid support, such as a glass slide, with multiple copies of each probe sequence attached within the same spot on the slide. Nucleic acid strands are extracted from the sample, fragmented and labelled with a fluorescent dye. The labelled strands are called targets. The targets are incubated with the array for 16–48 hours to allow hybridisation to occur [2-6]. The targets are excited with a laser and the resulting fluorescent signals from each of the spots are measured. Where more target strands have hybridised to the probes for a particular sequence, there will be a stronger fluorescent signal from the relevant spot. Hence, the signal intensity from the spot can be used to estimate the amount of the sequence in the sample [2-6].

Often, as in this study, competitive hybridisations are used. In this case, two targets, labelled with different dyes, are hybridised to the same array. The ratio of fluorescent signal intensities from the two dyes is measured, and used to estimate the ratios of the amounts of the target in each sample with the assumption that the targets have equal binding affinities to the probe [3,4].

However, microarrays are commonly used in situations where there may be mismatches between the probe and target sequences, such as variation between individuals, strain differences or interspecies differences. These sequence differences can reduce the hybridisation efficiency between probe and target strands, thus reducing the measured fluorescent signal intensity.

### Short oligonucleotide probes, cDNA probes and long oligonucleotide probes

Short oligonucleotide probes, such as Affymetrix 25 mer probes, are known to be very sensitive to mismatches [2,11-13]. This is partly due to the probe length and partly due to the analysis methods used [2,11-13]. Indeed, the observed sensitivity to mismatch position of Affymetrix 25 mer probes has been exploited for SNP detection in applications such as SNPscanner [15]. Long cDNA probes, often hundreds of bases long, are less sensitive to mismatches [16]. The usual explanation for this is that a single base mismatch is unlikely to have a substantial effect on the probe-target duplex melting temperature.

It might seem reasonable to assume that the effect of mismatches on long oligonucleotides, intermediate in length, would be intermediate between these two extremes. However, relatively few studies have examined hybridisation of mismatched targets to long oligonucleotide microarray probes. Kane and co-workers examined cross hybridisation of non-target DNA to 50 mer oligonucleotide expression arrays and found detectable hybridisation signals from non-target transcripts with similar sequence to the true targets or with a continuous stretch of sequence complementary to the probe. However, the precise effect of individual mismatches on signal intensities from long oligonucleotides was not investigated [17]. Letowski and co-workers investigated the influence of various factors on the performance of microarray probes of varying type and length, including 50 mer oligonucleotide probes that incorporated known mismatches. They reported that mismatches affected probe specificity, with mismatches at the ends of the probe having the least effect. Mismatches distributed along the length of the probe sequence caused more destabilisation of probe-target duplexes than mismatches clustered together [18].

In a review of genomic microarrays, Mantripragada and co-workers predicted that arrays using long oligonucleotides between 50 mers and 100 mers would largely replace BAC- and PCR-based microarrays for CGH [19]. Hughes and co-workers compared the performance of a range of inkjet-printed oligonucleotides and reported that 60 mers represented a practical compromise between maximum sensitivity and specificity [20]. Given the growing popularity of long oligonucleotide probes for both gene expression arrays [16] and CGH arrays, it is increasingly important to understand the effects of mismatches in reducing the signal intensity from these probes.

### Studying the effect of sequence mismatches using mouse CGH data

Mouse is an ideal species for investigating these effects, due to the availability of inbred strains and public datasets describing genomic sequence variation between these strains [14]. Long oligonucleotide CGH arrays provide a useful platform for examining the effect of mismatches since target abundances are largely fixed, target sequences will not be modified by alternative splicing and 60 mer oligonucleotides have been demonstrated to provide a good compromise between sensitivity and specificity [20,21].

We carried out competitive two sample hybridisations with dye-flip replicates for each of three inbred mouse strains (129P3/J, A/J and BALB/cJ) against a C57BL/6J reference on the Agilent whole mouse genome 244K CGH array and a custom 56K Agilent mouse CGH array, both using 60 mer oligonucleotide probes. We then compared NCBI mouse genome build 36 position information for the 8 million SNP in the public Perlegen dataset [14] and the Agilent probe sequences to identify SNP that would cause a mismatch between a probe and one or more of the test strain targets. Since the probes were designed against the C57BL/6 genome sequence any SNP would give rise to a mismatch between the probe and the test targets but not between the probe and the C57BL/6 control target and hence a higher signal from the control target if mismatches have an effect.

Our initial observations indicate a strong effect of mismatches on log_2 _signal ratio, dependent on the number of mismatches and on their position relative to the probe sequence. More specifically, we identified a strong correlation between log_2 _signal ratio and the maximum continuous length of complementary duplex when comparing probes overlapping 1 and 2 SNP.

## Results

In this study, the term "log_2 _signal ratio" refers to the base 2 logarithm of the signal intensity for the C57BL/6 reference sample divided by the signal intensity for the test sample (Equation 1).

(1) $${\text{log}}_{\text{2}}\text{ ratio}={\text{log}}_{\text{2}}\left(\frac{\text{reference normalised intensity}}{\text{test strain normalised intensity}}\right)$$

Thus, positive log_2 _signal ratios imply less efficient hybridisation for the test sample than for the C57BL/6 reference and conversely negative log_2 _signals imply more efficient hybridisation for the test sample. If the samples had equivalent levels of hybridisation to a probe, the log_2 _signal ratio would be close to 0.

We acquired log_2 _signal ratios for 235,389 probes on the whole genome array and 53,520 on the custom array. 15,286 (6.46%) of the whole genome array probes and 3,710 (6.93%) of the custom array probes overlapped one or more polymorphic loci in the Perlegen SNP set, representing a large, potentially powerful dataset we could use for examining the effect of mismatches on the signal ratios reported by 60 mer oligonucleotide probes. Table 1 lists the numbers of probes overlapping 1, 2 and 3 SNP for each test strain compared to C57BL/6J. All data for probes with mismatches in the custom and whole genome arrays is available in supplementary data [see Additional file 1 and Additional file 2]. A list of CNV called by the Agilent feature extraction software v9.5 is also available [see Additional file 3].

**Table 1:** Number and percentage of probes overlapping 1, 2 or 3 SNP loci for each test strain

**244 k whole genome probe set**	**1 SNP (% of probes in set)**	**2 SNP (% of probes in set)**	**3 SNP (% of probes in set)**
A/J v C57BL/6J	7782 (3.30)	779 (0.33)	35 (0.01)
BALB/cJ v C57BL/6J	7170 (3.04)	708 (0.30)	43 (0.01)
129P3/J v C57BL/6J	7967 (3.38)	864 (0.36)	41 (0.01)
Any v C57BL/6J	13683 (5.81)	1526 (0.64)	77 (0.03)
56 k custom probe set			
A/J v C57BL/6J	1343 (2.51)	120 (0.22)	5 (0.01)
BALB/cJ v C57BL/6J	1834 (3.43)	178 (0.33)	8 (0.01)
129P3/J v C57BL/6J	2273 (4.25)	233 (0.44)	11 (0.02)
Any v C57BL/6J	3415 (6.38)	352 (0.65)	16 (0.02)

### Mismatches are associated with reduced signal intensity from long oligonucleotide probes

**Figure 1:**
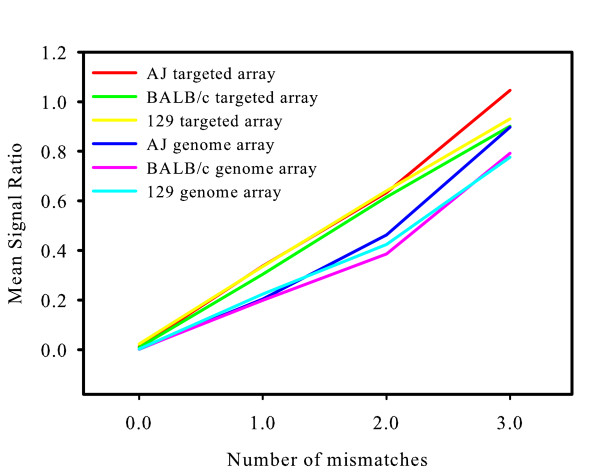
**Mean log_2 _signal ratio for probes containing each number of mismatches**. The trend of increasing mean log_2 _signal ratio with increasing number of mismatches is clear. The apparent increase in variance with increasing mismatch count is not significant and is likely to be a consequence of smaller numbers of probes with larger numbers of mismatches.

Known mismatches due to SNP in the test strains were associated with high positive mean log_2 _signal ratios, indicating that the signal intensity for the test strain samples was reduced. This effect is illustrated in figure 1, which shows the mean log_2 _signal ratios for all probes hybridizing to targets with 0, 1, 2 or 3 known mismatches.

There was a significant association between log_2 _signal ratio and number of known mismatches (ANOVA p < 0.001) and a significant correlation co-efficient between number of known mismatches and log_2 _signal ratio (r^2 ^= 0.94, p < 0.05).

The increase in mean log_2 _signal ratio with increasing number of sequence mismatches provides useful confirmation that sequence mismatches have an observable effect on signal intensities from long oligonucleotides as well as those from short oligonucleotides. It is clear that a higher number of mismatches is associated with a stronger effect on signal intensity. This observation might be expected, and could be consistent with a model of hybrid formation where the effect of mismatches simply results in loss of enthalpy from the hydrogen bonds that would have been formed during base-pairing.

### Mismatches near the middle of probes are associated with a greater reduction in signal intensity than those near the end of probes

**Figure 2:**
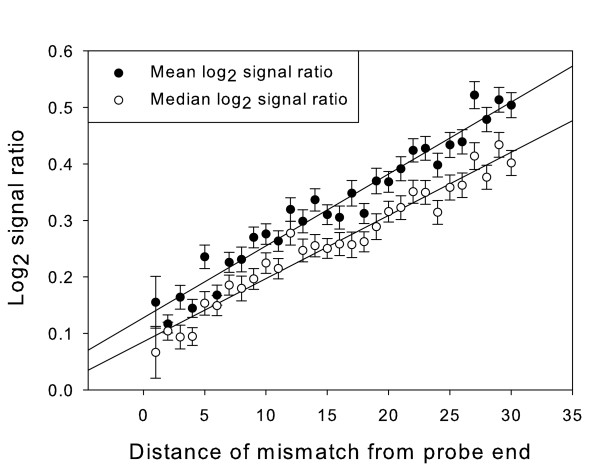
**Log_2 _signal ratio for duplexes containing 1 mismatch, in each possible mismatch position**. Mismatch position is measured in base pairs from the nearest end of the probe. The lines show the result of linear regression over the mean value for each position and the median value for each position. Error bars represent the standard error of the mean.

Known mismatches near the middle of probes are associated with higher average log_2 _signal ratios (and therefore greater reduction in test strain signal intensity) then those near the end of probes. Figure 2 displays a plot of average log_2 _signal ratio by mismatch position. The averages are over all probes hybridising to targets with one known single base-pair sequence mismatch in the relevant position, and the positions are counted from the end of the probe sequence nearest to the mismatch. A line was fitted to the scatter plot of log_2 _signal ratio against mismatch position, there was a strong correlation between mean log_2 _signal ratio and mismatch position (r^2 ^= 0.92) and the slope was significantly different from zero (F = 24.8, df = 35, p < 0.001).

The significant dependence of log_2 _signal ratio upon position of the single base mismatch was unexpected for long oligonucleotide probes. A correlation between log_2 _signal ratio and mismatch position would not be expected if the only effect of a mismatch was on enthalpy. The loss of enthalpy, from breaking of the 2 or 3 hydrogen bonds of a complementary base pair, is the same for all mismatch positions. However, the range and diversity of configurations that the probe and target strands can adopt also forms a contribution to the thermodynamic stability of the probe-target hybrid. Therefore the number of potential configurations, i.e. the entropy, must also be considered when attempting to understand the effect of sequence mismatches on log_2 _signal ratio.

### Log_2 _signal ratios are strongly correlated with the maximum length of perfect match

**Figure 3:**
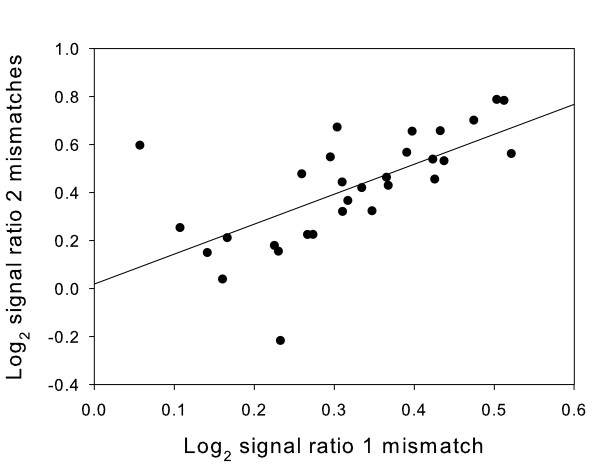
**Mean log_2 _ratio of probes with 1 and 2 mismatches paired by maximum length of perfect match**. Mean log_2 _signal ratios for probes with 1 or 2 mismatches from the whole genome array were obtained for each length of perfect match. Pairs of values were plotted against each other where they had the same length of perfect match. There was a significant correlation between the values from the two datasets (r^2 ^= 0.43).

If the length of perfect match between probe and target is a significant contributor to log_2 _signal ratio then a correlation would be expected between the signal ratios for probe-target pairs with 1 mismatch and pairs with two mismatches when they are compared based on the length of perfect match. Figure 3 shows a plot of mean log_2 _signal ratio of probes with one mismatch plotted against the mean of probes with two mismatches where both probes have the same length of perfect match. The Pearson correlation coefficient for the correlation between the probes with 1 mismatch and probes with 2 mismatches was 0.65 (r^2 ^= 0.43) indicating that length of perfect match accounts for approximately 43% of the variance in log_2 _signal ratio between probes with 1 and 2 mismatches. This suggests that for these long oligonucleotide probes and for small numbers of sequence mismatches the maximum length of perfectly matched duplex is a major determinant of the effect that sequence mismatches have upon log_2 _signal ratios. Consequently the observed increase in log_2 _signal ratio with increasing number of mismatches (Fig. 1) is largely due to the correlation between number of mismatches and length of perfect match.

These observations provide further evidence that the effect of mismatches is more complex than a simple loss of enthalpy for each mismatched base. It appears that a large factor in the effect of mismatches on 60 mer probe-target hybridisation is a reduction in the maximum length of perfectly matched duplex.

### Additional factors affecting the log_2 _signal ratio

**Figure 4:**
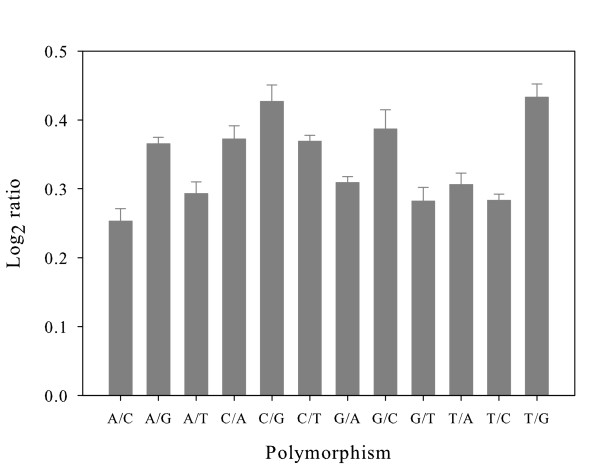
**Average log_2 _signal ratio for each possible transition or transversion**. The labelling for polymorphisms is the allele in the reference strain (C57BL/6J) followed by the allele in the test strain. E.g. A/C indicates that the reference strain has an A at the relevant position, and the test strain has a C. Since the probes are all designed to be complementary to the C57BL/6J reference, this means that there is an A-T base pair for C57BL/6J and a C-T pair for the test strain. Error bars represent the standard error.

Some polymorphisms might be more destabilising to the interaction between probe and target than others; either because they allow the formation of different numbers of hydrogen bonds in non-Watson-Crick base-pairing or because of other structural effects on the double-helix (for instance, two purines paired together might be so large compared to complementary pairs that they distort the double-helix). As expected, the log_2 _signal ratios for probe-target pairs containing mismatches varied for each different type of substitution. The mean log_2 _signal ratio for probes with different single mismatches on the mouse whole genome array varied between 0.25 and 0.43 (Fig. 4). The mean log_2 _signal ratio on the mouse whole genome array in the absence of mismatches was 0.01, so all polymorphism types were associated with a significant effect. The base changes that caused the largest effects were from pyrimidines to a G, which would lead to a GG or GA base pair. However, there is some indication that GA pairs can occur naturally and do not cause great instability [22]. In addition, a 2006 study by Wick and co-workers using short oligonucleotides (18 mers) found markedly different effects of each possible substitution to those we report here [23]. One possible explanation for this is that the effect of polymorphisms might be strongly influenced by the identity of the two neighbouring base pairs [24-26], which was not a factor included in either study. To compare the effect of the type of substitution to the effect of the position of the mismatch, we performed a two-way ANOVA of log_2 _signal ratios from the whole genome array data, with type of substitution and distance of mismatch from the probe end as explanatory factors. The majority (94.4%) of the total variation in log_2 _signal ratio was not accounted for by substitution type or mismatch position, revealing the intrinsically noisy nature of microarray data. Position of the mismatch accounted for nearly 4.6% of the total variation in contrast to 0.95% for the substitution type, i.e. the effect of mismatch position was almost five times larger than the effect of mismatch type. It should be emphasized that the small contributions of mismatch position and mismatch type to the variance in signal ratio does not mean that these effects are insignificant. The data in figures 1 and 2 show that the overall effect of mismatches is substantial, but the small contribution to the variance indicates that the effect of random noise on individual probes is highly variable. This estimate of the contribution of mismatches to the variance of log_2 _signal ratios is likely to be an underestimate due to the large numbers of unknown mismatches; less than 50% of SNP are included in the Perlegen dataset and indels are very poorly represented and were not incorporated in our study [14]. One group has suggested that >75% of polymorphic probes were caused by non-CNV differences such as SNP and simple repeat length variations [27]. However, the estimate of the relative contributions of substitution type and mismatch position should not be biased by missing data. The effects of random noise can always be reduced by replicated measurements, whilst the systematic effects due to mismatch position and type cannot. Most researchers would acknowledge the potential importance of substitution type for hybridisation strength, yet this analysis further confirms the dominant role of mismatch position, in particular perfect match length, in determining signal ratios.

GC content produces another effect related to the identity of bases in the sequence. Probes with a high GC content are known to have a higher melting temperature due to the presence of more hydrogen bonds. GC content may also affect probe specificity and the temperature sensitivity of probe-target hybrids [18]. However, probes are designed to have a similar melting temperature. In addition, since the observations of the position-dependent effect of mismatches were averaged over all probes, they should not be affected by GC content. To confirm that probe GC content did not have a significant effect on log_2 _signal ratio, we checked the correlation between proportion of GC bases and log_2 _signal ratio for all three test strains. As anticipated, the correlation coefficients were all close to zero (-4.84 × 10^-4 ^for A/J, -2.88 × 10^-3 ^for BALB/cJ and 3.2 × 10^-2 ^for 129P3/J).

**Figure 5:**
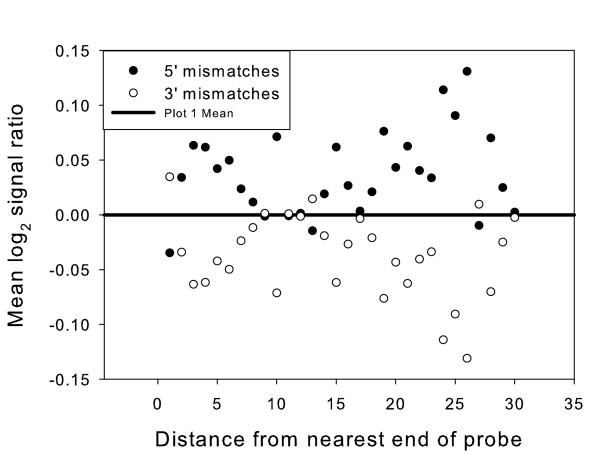
**Log_2 _signal ratios for probes with mismatches near the 3' or 5' end**. Average log_2 _signal ratio for probes with each length of perfect match, due to one mismatch closer to the 3' or 5' end of the probe. If there was no effect based on which end of the probe the mismatch was closer to, we would expect to see equal scatter above and below the identity line for mismatches near either the 3' or the 5' end. Probes with 3' mismatches are mostly above the line and probes with 5' mismatches are mostly below. Note that the 3' end of the probe is attached to the array surface.

Another, more subtle effect on log_2 _signal ratios was the position of mismatches relative to the 3' or 5' end of the probe. Mismatches near the 5' end of the probe were associated with higher log_2 _signal ratios than mismatches the same distance from the 3' (attached) end of the probe (Fig. 5). Although the mean difference in log_2 _signal ratio between 5' and 3' mismatches was only 0.07 this difference was highly significant (paired t-test, p = 9.7 × 10^-6^). This indicates that mismatches near the 5' end have a greater effect on hybridisation, and so produce a greater reduction in signal intensity from the probe. Our finding agrees with the results of a previous study using 60 mer oligonucleotides, which reported a greater effect of mismatches further away from the array surface, with the importance of a base to hybridisation efficiency described as being roughly proportional to the distance from the array surface. This effect was attributed to both steric and unspecified non-steric factors [20]. Studies on short oligonucleotides have identified a similar effect [23,28]. Wick and co-workers speculated that the probe-target duplex and dangling target ends near the array surface become partially immobilised in the DNA film, whereas further from the surface these could be more mobile, allowing the targets to dissociate and diffuse away from the array surface more easily when the duplex is destabilised by a mismatch [23].

Remaining possible sources of variations in the log_2 _signal ratios include random noise in the measured signal intensities, small deletions in the test strains and previously unidentified SNP (the Perlegen dataset is estimated to contain about 43% of SNP present in the strains genotyped [14]). Any of these causes might explain the probes in the dataset that produced a high positive log_2 _signal ratio but that did not overlap with any SNP locus in the Perlegen dataset. In order to determine whether unpublished SNP might contribute to non-zero log_2 _signal ratios the data was scanned to discover whether there was any significant excess of probes with log_2 _signal ratios > 1 in regions of the genome known to contain SNP in the relevant test strains. The mouse genome was divided into 50 kb blocks, then we obtained the number of SNP in each block for each of the test strains. There was a significant excess of probes with log_2 _signal ratios > 1 in the 50 kb intervals that had at least one SNP (χ^2^_1_; p < 10^-18^). There was also an excess of probes that had a log_2 _signal ratio > 1 and a SNP within 500 bp when compared with the same number of probes chosen at random. A/J and BALB/cJ probes with no mismatches in the Perlegen dataset but with a log_2 _signal ratio > 1 had a relative risk of 1.7 and 2.3 of having a mismatch within 500 bp compared with probes chosen at random (χ^2^_1_; p <10^-8^). For 129P3/J the relative risk was 1.2 (χ^2^_1_; p = 0.023). The presence of SNP that are informative between strains suggests that each strain carries a different form of the whole haplotype block that covers the region. The different forms of the haplotype may contain multiple SNP or small genomic indels that are not recorded in the Perlegen dataset and that might contribute to altered log_2 _signal ratios. This raises the possibility that probes with high log_2 _signal ratios might be used to identify candidate regions for re-sequencing to identify SNP and small CNV.

We also identified some probes with low negative log_2 _signal ratios, although only around 1/5 as many as those with high positive ratios. Possible explanations for negative log_2 _signal ratios include the presence of duplications in the test strains, deletions in the C57BL/6 reference DNA [27], SNP in the test strains that create additional probe binding sites and random noise in the measured signal intensities.

## Discussion

We have demonstrated that sequence mismatches are associated with higher log_2 _signal ratios from long oligonucleotide microarray probes. This effect is position dependent, with mismatches near the centre of a probe having a stronger effect on log_2 _signal ratio than mismatches near one end of a probe. There is a strong correlation between log_2 _signal ratios from probe-target pairs containing 1 mismatch and log_2 _signal ratios from pairs containing 2 mismatches when the pairs contain the same maximum length of perfectly matched duplex (r^2 ^= 0.43).

Whilst there is extensive evidence for an effect of mismatches on results from microarray hybridisations, much of this applies to results from short oligonucleotide arrays. Some studies have discussed an effect of mismatch position when using short oligonucleotide probes. Terminal mismatches in very short duplexes have long been known to have less effect than internal mismatches [29]. Mismatches near the centre of the probe have a stronger destabilising effect than mismatches close to either end, both for hybridisations in solution [5] and for microarray hybridisations [13,23,28,30]. However, while this difference in destabilisation has been observed frequently, and used in applications such as SNP detection [15,28], the difference has not been examined in detail or explained in terms of thermodynamic properties.

Comparatively fewer studies have reported mismatch effects on results obtained using long oligonucleotide probes [17,18,20]. Hughes and co-workers described the importance of a base in terms of microarray hybridisation efficiency as roughly proportional to the distance of the base from the array surface, possibly due to steric effects [20]. Letowski and co-workers identified a smaller destabilising effect for mismatches clustered at either end of a probe than for mismatches clustered near the probe centre, and likewise a smaller effect for mismatches clustered in any position than for mismatches spread out along the probe sequence [18]. However, they did not attempt to explain this finding and it is clear that the dependence of duplex stability on the maximum length of perfect match in the probe-target hybrid might provide such an explanation.

### The role of maximum perfect match length

We found that, at least for small numbers of sequence mismatches, the mismatch positions themselves are less important than the maximum length of perfect match that results from the mismatches. For one mismatch the length of perfect match also appears to exert a greater influence on log_2 _signal ratios than the type of polymorphism, accounting for nearly five times as much of the variation in log_2 _signal ratio.

There is some support for the suggestion that maximum length of perfect match has a role in determining hybridisation efficiency. Kane and co-workers examined cross-hybridisation of non-target DNA to 50 mer oligonucleotide expression arrays. Detectable signals were found from non-target transcripts that contained a continuous region of 15 bases or longer perfectly matched to the probe sequence and longer continuous complementary regions were found to produce a stronger signal [17]. Sasaki and co-workers identified a similar effect on hybridisation of full-length cDNA targets to tiling arrays of Affymetrix 25 mer genomic probes [31]. However, none of these studies investigated the effects of individual single base mismatches, and although the effect has been observed, there has not been a systematic investigation or an explanation of why this effect occurs.

**Figure 6:**
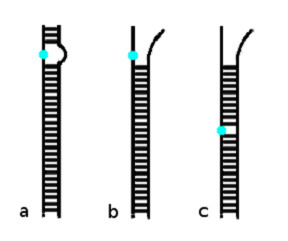
**Alternative scenarios for probe-target binding configurations**. a. a configuration where the duplex is fully-formed. A mismatch might cause some loss of hydrogen bonding at the mismatch position, and possibly at a small number of adjacent base pairs, indicated by the loop. If all of the probes were in this type of configuration, mismatch position would not be expected to have any significant effect. b. a configuration where the duplex is partially formed. A mismatch that occurs in a region where the duplex is not formed has no effect on the stability of the hybrid. This is more likely for mismatches near the ends of the probe. c. the same configuration as 6b, but in this case the mismatch is near the centre of the probe, and so lies within the duplex region and will have some effect on the stability of the hybrid (this might be the loss of a few hydrogen bonds, as in figure 6a, a total disruption of the duplex or some intermediate scenario). *These figures are intended as schematic diagrams of some possible duplex configurations, not as detailed representations of any particular duplex*.

If the effect of a sequence mismatch was simply the loss of enthalpy generated by the 2 or 3 hydrogen bonds formed in a complementary base pair, then the position of the mismatch in the duplex would not be expected to have any strong influence. The fact that we observed a strong dependence on mismatch position suggests that the entropic contribution to the thermodynamic stability is important, and that careful consideration of the many different configurations in which a target strand can bind to a probe strand is required. At the relatively high temperatures recommended for microarray hybridisations [5], in particular the elevated temperatures used for long oligonucleotide arrays [7-9,20,21,32], the vast majority of probe-target hybrid configurations will not represent full length duplexes, but partial duplexes with dangling ends, as indicated in figures 6b and 6c, or local areas of disorder due to loops, as indicated in figure 6a. Within the modern helix-coil transition theory of DNA melting introduced by Poland and Scheraga [33] loop configurations are downweighted, relative to dangling end configurations, by a factor σ, the co-operativity factor. Typically σ is in the order of 10^-5^. Therefore, for oligomers up to a few hundred base-pairs the effect of loops on hybrid stability can be safely ignored and one can focus solely upon the dangling end configurations [34]. If the sequence mismatch occurs towards one end of the probe strand then most hybrid configurations, with only partially formed duplexes, will be unaffected. Therefore, the free energy for probe-target hybrids in the presence of a single sequence mismatch will largely be similar to that when no mismatch is present, i.e. introducing a sequence mismatch near the ends of the probe has only a marginal effect upon the thermodynamic stability of probe-target hybrids. Conversely, if the sequence mismatch occurs towards the middle of the probe strand, a larger number of hybrid configurations are affected, leading to a greater reduction in hybrid stability.

### Theoretical model development

We have begun development of a theoretical model of microarray hybrid formation, based upon the Poland-Scheraga model [33], that explicitly takes into account partial duplex configurations (as outlined above). The current version obtains good agreement with the qualitative aspects of the experimental results presented here. Generally, this highlights the need to build upon existing models of hybrid formation and take into account the specific conditions unique to microarrays. Several research groups have found hybridisation behaviour on microarrays to differ from that in solution, with attachment to a surface having a marked effect [13,24,29,30], though for short oligonucleotide microarrays, hybridised at relatively low temperatures, there are strong correlations between microarray intensities and the free energies for the same probe-target duplexes in solution [26,30,35] and between the cost of mismatches for microarray probes and the cost calculated in solution [24]. It is worth noting that entropic contributions to free energy changes on arrays are obviously different to those in solution [35], due in part to the additional complexities involved in hybridisation of targets to microarray probes, such as the probes being attached at one end to a surface and probes being closely spaced on the array [36-39].

### Future directions

Even without development of new models, these results have implications in terms of microarray design and interpretation of microarray results. Most current approaches to microarray design are based on data from hybridisations in solution [23,30], which may not accurately reflect the hybridisation conditions for microarrays. As the potential applications for microarrays extend, there is an increasing need to understand the effects of sequence mismatches. Several studies have demonstrated that oligonucleotide arrays can be used for genomic DNA capture for high-throughput sequencing of specific genomic regions [7-10]. For example, it is possible that this approach could be used in attempts to re-sequence all human coding regions in hundreds or even thousands of individuals, providing a resource for investigating links between disease susceptibility and genetic variations [7,10]. The evidence presented in this study suggests that probes for DNA capture should be designed to avoid SNP loci. If that is not possible, then positioning SNP to maximize the length of continuous perfect match to targets is likely to reduce the risk of selectively capturing only some of the intended target strands.

Our results also raise the possibility of using microarray CGH results to identify putative small CNVs and SNP for confirmation by high-throughput sequencing or other methods. CNVs are an important type of genetic variation. Approximately 4% of the human genome has undergone recent duplications [40-42]. CNV have also been identified between different mouse strains, and even between different colonies of the same inbred mouse strain [27,43]. Studies of murine CNV have alluded to thousands of single-probe aberrations, which were attributed to the presence of SNP [27,44]. Microarray CGH analysis software usually requires 3 continuous probes passing a log_2 _signal ratio threshold in order to call an aberration. However, results from human ultra-high-density tiling arrays find many small CNV < 1000 bp [45]. Egan and co-workers investigated 65 single probe aberrations in a comparison of C57BL/6 mice from different colonies. 20 of these were successfully confirmed as small CNV and a further 11 were found to contain SNP but all these would be missed by a heuristic that required 3 contiguous probes to have a non-zero signal ratio [27]. In this study, we showed a large excess of probes with log_2 _signal ratio greater than 1 or less than -1 in more than one strain over what would be expected by chance, suggesting some potential for these single probe aberrations to indicate putative SNP or small CNV.

## Conclusion

Sequence mismatches have an observable effect in reducing the signal intensity reported by long oligonucleotide probes on CGH microarrays. This effect depends on the position of the mismatch relative to the probe, being stronger for mismatches near the centre of the probe than for those at the ends. We also found that the length of perfect match can have a stronger effect on log_2 _signal ratios than the type of polymorphism. These observations have implications in terms of array design and analysis, relevant to the use of microarrays in genomic DNA capture for high-throughput sequencing.

## Methods

### Microarray CGH data

We obtained genomic DNA from Jackson Laboratories (Bar Harbor, Maine, USA) for mouse strains C57BL/6J (Jackson stock number 000664), BALB/cJ (000651), 129P3/J (000690) and A/J (000646).

We carried out two-sample hybridisations, using C57BL/6J as a reference, using the Agilent Mouse Genome CGH Microarray 244A platform and a custom 56K Agilent mouse microarray platform. Both platforms use inkjet-printed 60 mer oligonucleotide probes [46].

We performed one hybridisation plus one dye-flip replicate for each of the three test strains (129P3/J, A/J and BALB/cJ) on each of the two array platforms. We hybridised 12 μg gDNA in 520 μL of 750 μM NaCl at 65°C for 48 hours, followed by two 5 minute washes at 37°C, according to manufacturer's instructions [32].

We used the Agilent feature extraction software to carry out a linear dye adjustment using a calibration sample of probes, equivalent to a centering normalization protocol [47], according to the standard procedures described in the Agilent feature extraction software v9.5 reference guide [48]. The inclusion of dye-flips within our experimental design effectively automatically implements a paired slide normalisation to produce centralised log_2 _signal ratios of test strain to C57BL/6J and eliminates intensity-dependent bias within the log_2 _signal ratios [49]. We then used Z-scoring to identify aberrant regions, following the standard Agilent procedures described in the CGH Analytics 3.4.40 user guide [50].

### Combining probe sequence and SNP data to identify mismatches in probes

We retrieved SNP data from the Perlegen dataset (genotypes for 8 million polymorphic loci from resequencing 15 inbred mouse strains, commissioned by the National Institute of Environmental Health Sciences) [14].

We obtained probe sequences and positions on the NCBI34 mouse assembly from Agilent and mapped probes onto a local copy of the NCBI36 mouse assembly using BLASTn. Probe information is available from GEO [1] along with other array data under accession [GEO:GSE9669]. We discarded probe sequences without a high-scoring match (e-value < e^-17^) the same length as the probe and did not include them in the dataset. We conducted BLASTn searches against the whole genome build for a 85805 probe sample. Only 1 probe had perfect BLASTn matches on more than one chromosome. For the remaining probes, we only performed BLASTn searches against the chromosome listed in the Agilent annotation. 699 out of over 235000 probes (0.28%) had perfect matches with more than one region of the same chromosome.

We extracted positions of SNP within the NCBI36 mouse assembly from the Perlegen annotation and used them to identify the positions of mismatches within each probe. A table of probes that contained SNP together with SNP position, substitution type, length of perfect match and log_2 _signal ratio is included in supplementary data for the mouse whole genome array [see Additional file 1] and supplementary data for the custom array [see Additional file 2].

### Data handling and analysis

We developed a MySQL database to store the positions of probes and of mismatches between C57BL/6J and each test strain (129P3/J, A/J and BALBc/J) to facilitate analysis. We wrote Perl scripts to make comparisons and calculations using this data, such as counts of the number of probes over various thresholds and statistical tests. The Perl scripts and the database tables are available from the authors upon request.

## Abbreviations

CGH: Comparative Genomic Hybridisation; CNV: Copy Number Variation; SNP: Single Nucleotide Polymorphism.

## Authors' contributions

CR reanalysed the data, drafted the introduction and discussion and rewrote the results. HAN initiated the study, carried out the primary analysis and drafted the results. AB proposed that the effect of mismatches was associated with length of perfect match. SJK participated in the design of the study. HH did the primary data extraction and advised on the analysis. DCH identified the relative roles of enthalpy and entropy, participated in the analysis and drafted parts of the discussion. All authors read and approved the final manuscript.

## Supplementary Material

Additional file 1 contains a table of all the probes that contained mismatches in the whole genome array. This table includes the position of the mismatch(es) within each probe and the signal ratio for each test strain. Click here for file

Additional file 2 contains a similar table for the custom array. Click here for file

Additional file 3 contains a table of CNV that were identified by the Agilent feature extraction software v9.5. Click here for file
